# Seasonal Evaluation of Freshness Profile of Commercially Important Fish Species

**DOI:** 10.3390/foods10071567

**Published:** 2021-07-06

**Authors:** Patrícia G. Cardoso, Odete Gonçalves, Maria F. Carvalho, Rodrigo Ozório, Paulo Vaz-Pires

**Affiliations:** 1 CIIMAR—Interdisciplinary Centre of Marine and Environmental Research, Terminal de Cruzeiros de Leixões, Av. General Norton de Matos, S/N, 4450-208 Matosinhos, Portugal; odete007@gmail.com (O.G.); mcarvalho@ciimar.up.pt (M.F.C.); rodrigo.ozorio@ciimar.up.pt (R.O.); vazpires@icbas.up.pt (P.V.-P.); 2 ICBAS—Abel Salazar Institute for the Biomedical Sciences, University of Porto, R. Jorge Viterbo Ferreira, 228, 4050-313 Porto, Portugal

**Keywords:** freshness profile, seasonal evaluation, quality variations, wild fish, farmed fish, spoilage bacteria, analytical methods

## Abstract

Freshness is considered one of the most important parameters to judge the quality of most fish products. In the current study, the seasonality effect on the freshness profile of different economic fish species was evaluated for the first time, using three different approaches (sensory: Quality Index Method (QIM) and European (EC) Scheme; physical: Torrymeter (TRM) values; and microbiological analyses: Total Viable Counts (TVC) and degradative bacteria). Over a year, individuals of farmed fish *Sparus aurata* and *Dicentrarchus labrax*, as well as the wild fish *Trachurus trachurus*, *Scomber colias*, and *Sardina pilchardus*, were sampled seasonally for the evaluation of their freshness profile over 10 days on ice. In general, data showed an increase in QIM values, a decline in TRM, and an increase of spoilage bacteria throughout the storage time, revealing a clear temporal degradation of the quality of the fish. Additionally, some signs of seasonality effect could only be observed for some species. For example, the seabass *D. labrax* showed lower numbers of degradative bacteria in winter than in the other seasons, suggesting a high potential to be marketed in a fresher condition, especially during that season. On the other hand, *S. colias* showed higher freshness scores (i.e., higher TRM values in spring and autumn and lower numbers of bacteria in summer) from spring to autumn. However, from the five studied species, *S. colias* presented the lowest freshness values, indicating a higher fragility of this species. This information is extremely relevant for consumers and retailers that want to invest in higher quality products, as they would thus be able to choose certain species in detriment of others. Additionally, obtained data showed that farmed species reached day 10 of storage time with lower values of QIM and microbial counts (cfu), as well as higher values of TRM, in relation to wild species. These results reinforce the idea that farmed fish can, under proper conditions, present high quality/freshness profile.

## 1. Introduction

Fisheries and aquaculture production have a significantly lower carbon footprint compared to that of chicken, pork, and beef production. In fact, fish has rapid growth and relatively low production cost, contributing to a healthy diet due to its high-quality protein, other essential nutrients such as omega 3-fatty acids, and low-fat content. Due to these characteristics, fish products have shown increasing demand, with a contribution of 60% of the world protein supply. In addition, 60% of developing countries obtain at least 30% of their animal protein from fish products [1]. The quality of fish is a major concern for producers, retailers, and consumers, with the last being increasingly demanding for high quality products. Freshness is a property that significantly affects the fish quality; it is probably the most important criterion to judge the quality of most of fish products [2]. The other three pillars defined by [3] are safety, traceability, and authenticity. One of the parameters that can greatly affect fish freshness is seasonal variation, which directly influences fish chemical composition [4]. For example, culture temperature may affect the amount of deposited lipids, as well as the fatty acid profile. These changes could affect the nutrient demands of the fish, as well as their organoleptic attributes [5]. Considering that fish is highly perishable (among other reasons, due to their fragile fatty acid composition, high content of water, and endogenous enzymes), loss of quality/freshness is very rapid and depends on a complex process where physical, chemical, and microbiological reactions are involved [1].

Most of the methods used to assess fish freshness are based on sensory analysis, so this kind of evaluation is considered one of the most effective. In the last two decades, several spectroscopic techniques have been successfully applied to determine the freshness of fish and/or meat in a rapid and non-destructive way. Some examples are infrared, fluorescence [6], nuclear magnetic resonance, and Raman spectroscopies, as well as some instrumental sensors, such as the electronic nose [7]. These techniques are fast, low cost, environmentally friendly, usually require little or no sample preparation, and allow one to avoid sample destruction [8,9,10]. Despite the mentioned advantages, the utilization of spectroscopic techniques in real-life applications is still limited. Meanwhile, the classical methods, based on the four specific fields—sensory, chemical, physical, and microbiological—are still the most used in companies and laboratories.

Regarding sensory analysis, the quality index method (QIM) is now widely used for this purpose. It is based on the evaluation of modifications of relevant characteristics of seafood, such as the skin, eyes, gills, and odor. It uses the attribution of demerit points in a scale from 0 to 3 (most-less fresh) [11]. It is a simple, fast, economic, and non-destructive grading system for fish freshness evaluation, but it is somewhat subjective. Thus, in order to minimize errors, it is important for it to be used by trained people. Another sensory method for fish quality assessment is the UE Freshness Grading (or EC scheme), presented for the first time in Council Regulation no. 103/76. There are three levels in the EC scheme: E (Extra, the highest quality), A (good quality), and B (satisfactory quality). Below level B (sometimes called Unfit or C), fish is not acceptable for human consumption, so it is discarded or rejected [12].

As no single method is sufficient to determine the freshness/quality of fish products, sensory parameters should be complemented by other objective techniques, such as physical and microbiological ones [13]. Physical analysis includes the measurement of dielectric muscle properties through the use of dedicated equipment, such as the Torrymeter [14]. The principle for electrical measurements of fish is based on the fact that cell membranes in fish muscle tissue are progressively disrupted by enzymatic degradation, which leads to a decline in electric resistance, so electric impulses become lower with time [13,15].

The microbiology associated with the different aquatic species reflects the bacterial population of the adjacent environment and the manipulation/processing conditions. In recently processed fish, specific spoilage organisms (SSO) occur in low numbers, but the number of SSO also increases as the time storage increases and is responsible for the rejection of specimens. In initial conditions immediately after catch, individuals can present total viable counts of 10^1^–10^4^ colony-forming units (cfu)/g or cm^2^, while values can range between 10^7^ and 10^9^ cfu/g or cm^2^ at the end of an individual’s shelf-life [13].

The main goal of this work was to evaluate the seasonal pattern of the freshness of commercially important fish species (e.g., wild—Atlantic horse mackerel, and Atlantic chub mackerel; farmed—gilthead seabream and European seabass) in order to identify freshness variations between different product origins and seasons. This will allow for the suggestion of the best fish product to retailers and consumers in a specific season and, at the same time, will contribute to the better management of stocks by retailers and to a reduction of returned products. For the accomplishment of this study, complementary sensory, physical, and microbiological analyses were carried out.

## 2. Materials and Methods

### 2.1. Animals

For this work, five fish species—three wild species (*Sardina pilchardus*, *Trachurus trachurus*, and *Scomber colias*) and two farmed ones (*Sparus aurata* and *Dicentrarchus labrax*)—were selected. These species were selected because they are the most consumed in Portugal, according to information from the biggest retailers. For the wild fish, a total number of 48 sardines (*Sardina pilchardus*) with average length of approximately 14.86 cm (±2.47), 96 Atlantic horse mackerels (*Trachurus trachurus*) with average weight of approximately 200 g (±1.47), and 96 Atlantic chub mackerels (*Scomber colias*) with average weight of approximately 300 g (±1.80) were analyzed. Regarding farmed fish, 96 gilthead seabream (*Sparus aurata*) and 96 European seabass (*Dicentrarchus labrax*) with average weight of approximately 250 g (±1.19) were used. The farmed species (*S. aurata* and *D. labrax*) were obtained from the company Pescódromo de Lavos at Figueira da Foz, Portugal, and the wild fish were obtained from Docapesca, Portos e Lotas, SA (Delegation of Matosinhos), both in the northern region of Portugal. All the fish were sampled over a year, from March 2019 to February 2020 across the four seasons (spring, summer, autumn, and winter), except for the sardines that were only collected in specific summer and autumn months. For this reason, results from the sardines were not integrated in the seasonal analysis, and it was decided to present them in the Supplementary Materials.

### 2.2. Fish Storage

During each season (spring, summer, autumn, and winter), two sampling campaigns were performed. For each campaign, 12 individuals of each species were selected and immediately stored in ice at 0–4 °C for 10 days in self-draining boxes. The ice was added as needed during the storage. At day 1 (D1—24 h after death), day 4 (D4), day 7 (D7), and day 10 (D10), 3 individuals were collected from the boxes and separately analyzed.

### 2.3. Sensorial Analysis (Organoleptic Parameters)

Sensory evaluation was done through the QIM [7] and EC Freshness Grades (EC) scheme [16]. Both methods were individually applied for all the fish at each sampling day. For the studied species, quality attributes for appearance/texture, eyes, gills, skin, and anal area were analyzed along with degradation time according to the QIM scheme (from D1 to D10). Every quality attribute was evaluated according to descriptions, ranging from 0 (most fresh) to 3 (less fresh), and the final value corresponded to the sum of the scores of the different attributes [13,17]. For the case of the EC scheme, each quality attribute was evaluated according to descriptions as “Extra”, “A”, “B”, or Rejected [16]. The freshness profiles of fish from the “Extra” category range from 3 to 2.7, those from the “A” category range from 2.7 to 2, and those from the “B” category are between 2 and 1, according to Council Regulation n° 103/76.

### 2.4. Physical Analysis

To evaluate the dielectric muscle properties, a Torrymeter (TRM 295, Distell, West Lothian, Scotland, UK) was used according to the equipment user manual.

The anterior-dorsal area was selected for measurements in all fish. For each fish, 8 readings (4 in each side) were done. Residual ice was cleared from the surface, and both sides were analyzed. The electrodes were cleaned between measurements, protected with cling film, and placed on ice to maintain the temperature around 0 °C. This procedure allowed us to maintain the electrodes at a temperature similar to the temperature of the fish skin without damaging the equipment.

### 2.5. Microbiological Analysis

Skin mucus (23.7 cm^2^ for *S. aurata* and *D. labrax*, 15.9 cm^2^ for *T. trachurus* and *S. colias*, and 3.14 cm^2^ for *S. pilchardus*) was swabbed with sterile cotton swabs at D1, D4, D7, and D10 (*n* = 3 fish/day). Swabs were then transferred to tubes containing 2 mL of a 0.9% saline solution that were vigorously vortexed to release the attached microorganisms. Appropriate serial decimal dilutions were done and inoculated in duplicate a Lyngby in iron agar solid medium using the drop (20 µL) method. Total viable counts (TVC) and selective counts of H_2_S-producing bacteria were accomplished after 48 h of incubation at 20 °C. Colony counts were performed in duplicate and presented as the logarithm of cfu/cm^2^.

### 2.6. Data Analyses

Two-way ANOVA (factors: time × seasons) was applied to evaluate differences in freshness parameters (i.e., QIM, EC scheme, TRM, and microbiology). All data were checked for normality (Kolmogorov–Smirnov test) and homogeneity of variances (Levene’s test) [18]. When the assumptions were not accomplished, a non-parametric 2-way ANOVA on ranks was applied. This analysis was performed with the Statistica7 software.

Then, data for each species were log-transformed, converted in a similarity matrix (using Euclidean distance), and analyzed with PERMANOVA for the factors of time and season, as well as with a PCO to visualize differences between treatments [19]. This multivariate analysis was performed with the PRIMER v6 and PERMANOVA software [19].

## 3. Results

### 3.1. Farmed Species

#### 3.1.1. Gilthead Seabream

**Figure 1:**
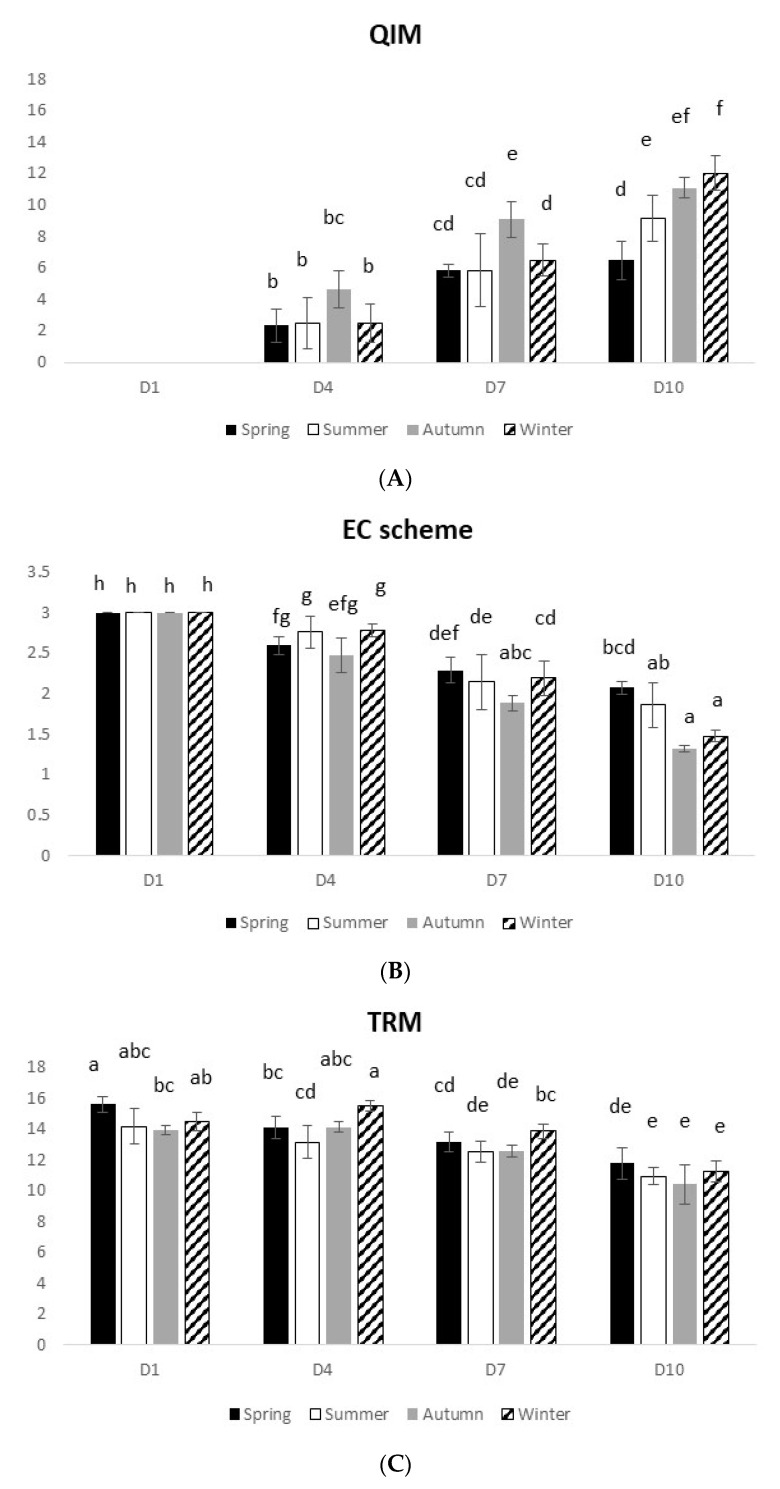

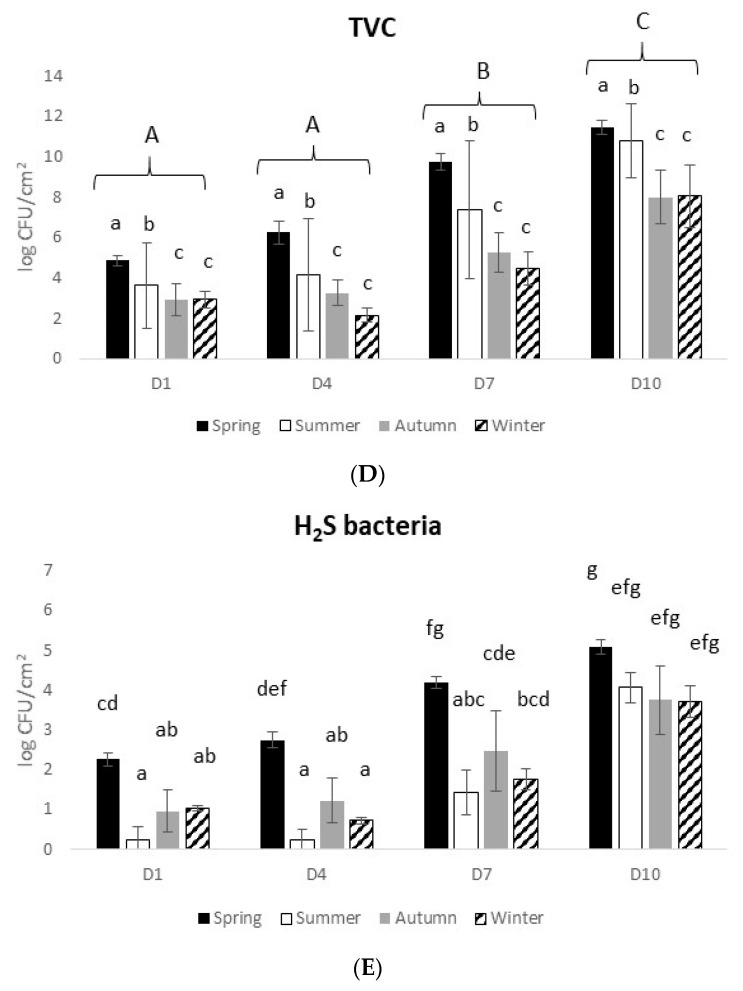
Seasonal gilthead seabream freshness profile over 10 days on ice, assessed by different methods: (**A**) Quality Index Method, (**B**) EC scheme, (**C**) Torrymeter (TRM), (**D**) microbiology (TVC), and (**E**) microbiology (H_2_S-producing bacteria). Values represent mean ± SD (*n* = 6). In Figure 1D, different uppercase letters indicate significant differences among days, while different lowercase letters indicate significant differences among seasons. In the other figures, different letters indicate significant differences among treatments.

In terms of sensory analysis, both methods (i.e., QIM and EC scheme) presented similar results, i.e., an expected loss of freshness profile with time (Figure 1A,B). At D10, QIM ranged from 6.5 to 12 (spring–winter), while the EC scheme ranged from 2.0 to 1.5 (spring–winter). A significant interaction between time and seasons was observed for both parameters (QIM: 2-way ANOVA on ranks, F_(9,80)_ = 6.056 and *p* = 0.000002; EC scheme: 2-way ANOVA on ranks, F_(9,80)_ = 3.44 and *p* = 0.001).

Concerning the physical analysis, measured with the Torrymeter (TRM), a decline of the Torrymeter values with time, meaning a decline of fish freshness, was observed (D1–D10: 14–11) in accordance with the previous methods (Figure 1C). Additionally, a significant interaction between both studied factors was observed (TRM: 2-way ANOVA on ranks, F_(9,80)_ = 3.61 and *p* = 0.0008).

Finally, the microbiological analysis of the fish skin showed significant differences among sampling times (2-way ANOVA on ranks, F_(3,80)_ = 71.34 and *p* < 0.05); the TVC ranged from 10^5^–10^3^ (D1, spring–winter) to 10^11^–10^8^ cfu/cm^2^ (D10, spring–winter) (Figure 1D). A significant decline in TVC from spring to autumn/winter was observed (2-way ANOVA on ranks, F_(3,80)_ = 29.49 and *p* < 0.05). The total number of H_2_S-producing bacteria significantly increased with time (Figure 1E), and an interaction between time and season was observed (2-way ANOVA on ranks, F_(9,68)_ = 2.93 and *p* = 0.005).

#### 3.1.2. European Seabass

**Figure 2:**
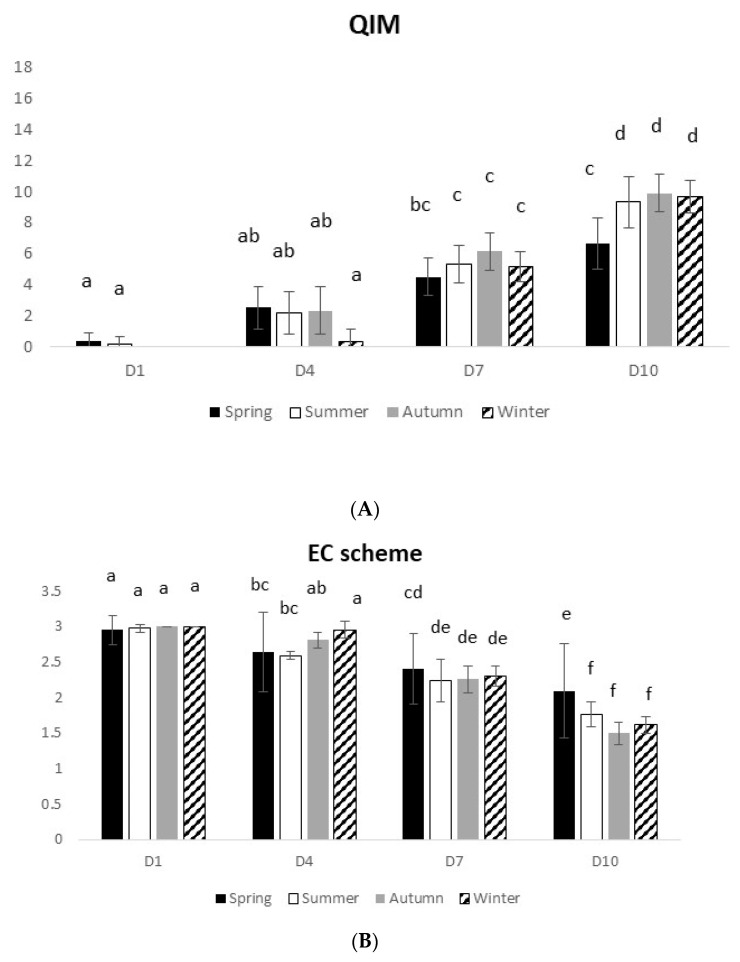

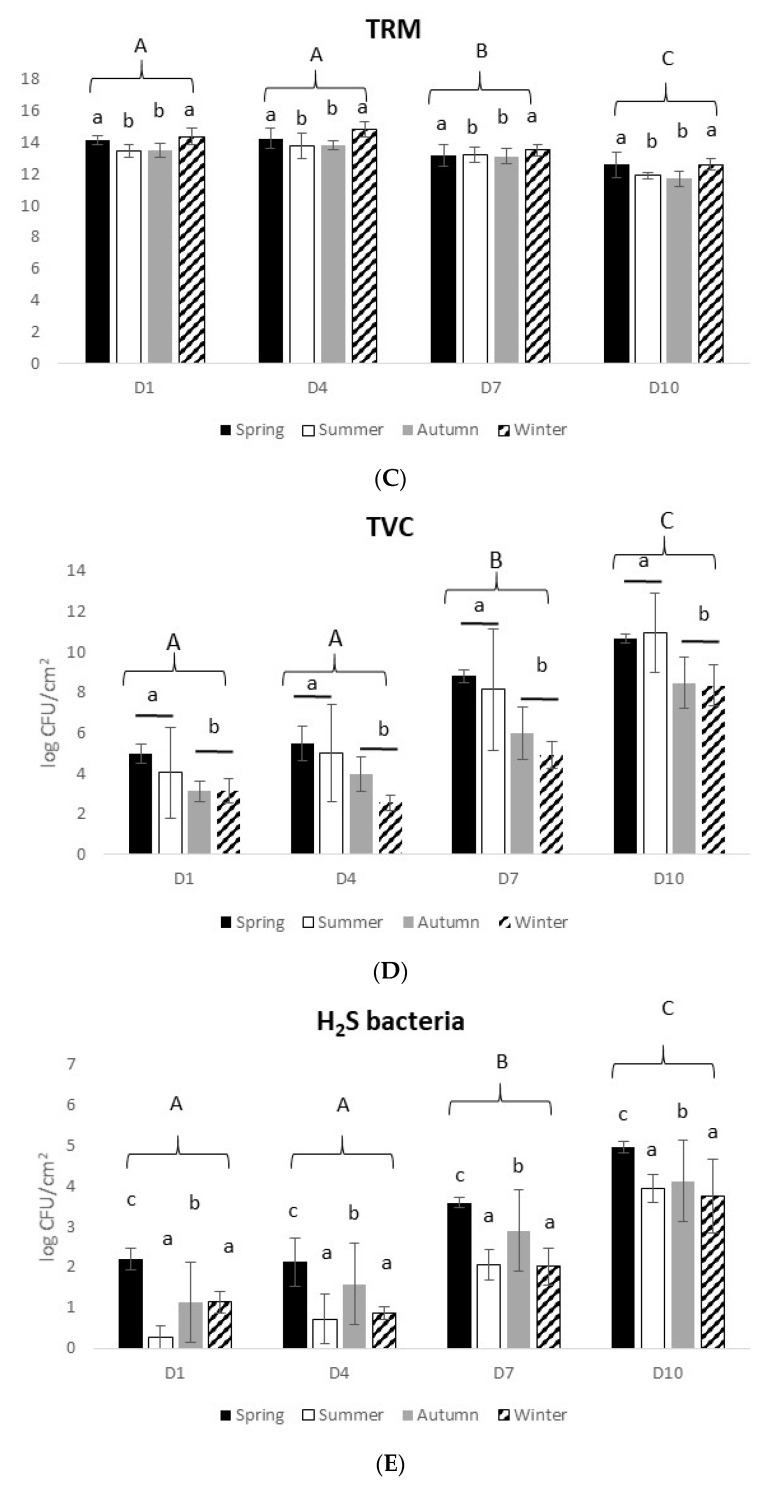
Seasonal seabass freshness profile over 10 days on ice, assessed by different methods: (**A**) Quality Index Method, (**B**) EC scheme, (**C**) Torrymeter (TRM), (**D**) microbiology (TVC), and (**E**) microbiology (H_2_S-producing bacteria). Values represent mean ± SD (*n* = 6). In Figure 2C–E, different uppercase letters indicate significant differences among days, while different lowercase letters indicate significant differences among seasons. In the other figures, different letters indicate significant differences among treatments.

The results of the sensory analysis in European seabass were similar to those observed in gilthead seabream. The QIM and EC scheme methods were consistent, indicating a decline of fish freshness with time (Figure 2A,B). At D10, QIM ranged from 6.7 to 9.7 and EC scheme ranged from 2.1 to 1.6 (spring–winter). A significant interaction between time and seasons was observed for both methods (QIM: 2-way ANOVA on ranks, F_(9,80)_ = 3.43 and *p* = 0.001; EC scheme: 2-way ANOVA on ranks, F_(9,80)_ = 8.57 and *p* = 0.00).

The physical analysis revealed a significant decline of fish freshness with time (2-way ANOVA, F_(3,80)_ = 70.53 and *p* = 0.0), reaching D10 with values ranging from 11.7 to 12.5 (Figure 2C). The summer and autumn presented lower freshness values than spring and winter (2-way ANOVA on ranks, F_(3,80)_ = 13.46 and *p* = 0.0).

The TVC in the skin significantly increased throughout time (2-way ANOVA on ranks, F_(3,80)_ = 80.36 and *p* = 0.0), from 10^5^–10^3^ (D1, spring–winter) to 10^11^–10^8^ cfu/cm^2^ (D10, spring–winter) (Figure 1D). Significant differences among seasons were also detected (2-way ANOVA on ranks, F_(3,80)_ = 20.36 and *p* = 0.0), in which total bacteria in spring/summer were higher than in autumn/winter. Additionally, a significant increase in the number of degradative bacteria with time (2-way ANOVA on ranks, F_(3,68)_ = 120.04 and *p* = 0.0) was observed (Figure 2E). Regarding the seasonal profile, some significant differences were detected in terms of number of degradative bacteria, with summer and winter presenting lower numbers of H_2_S bacteria than spring and autumn (2-way ANOVA on ranks, F_(3,68)_ = 30.99 and *p* = 0.0).

### 3.2. Wild Species

#### 3.2.1. Atlantic Horse Mackerel

**Figure 3:**
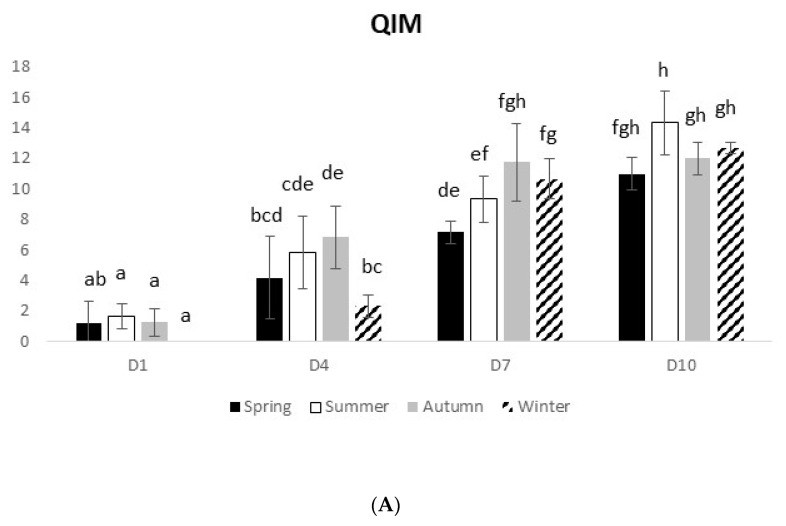

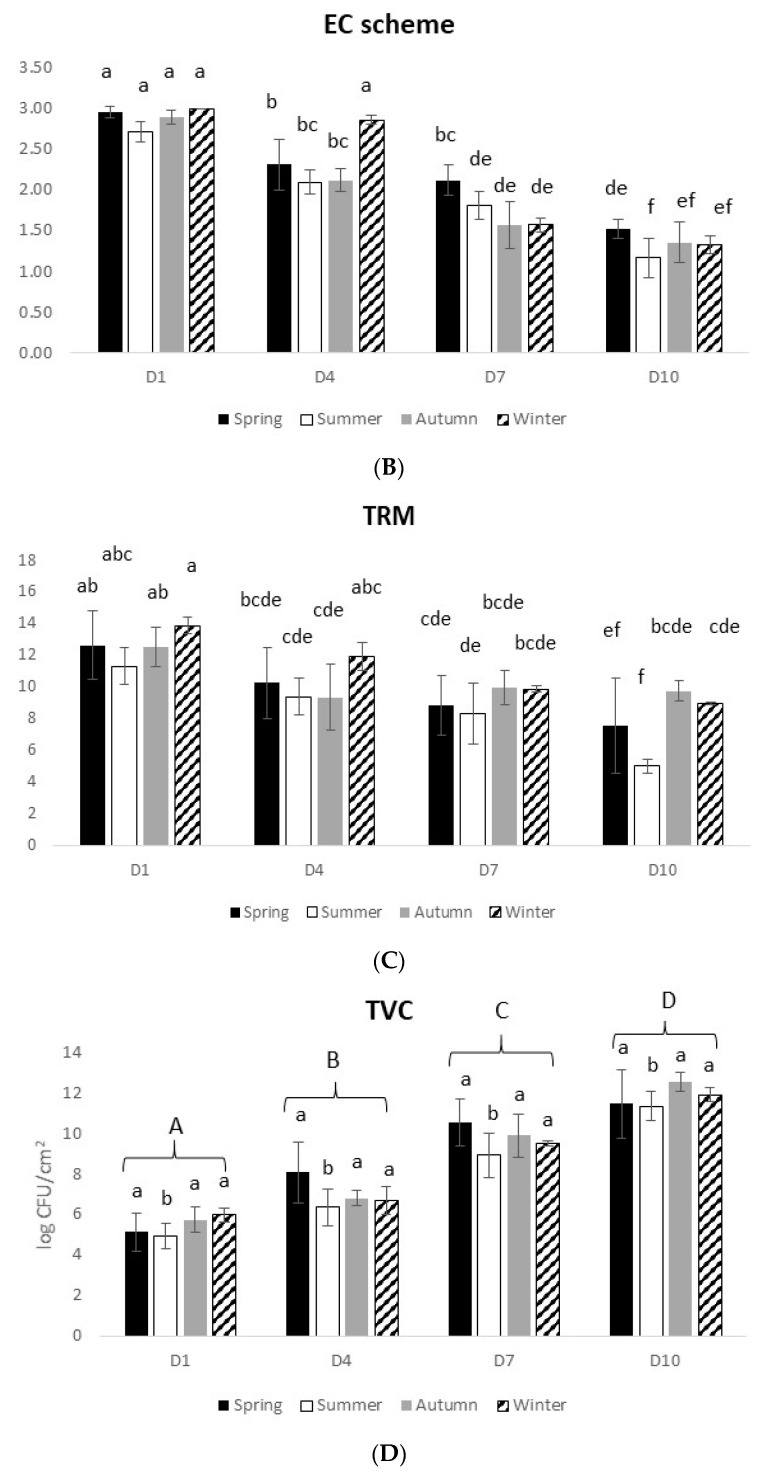

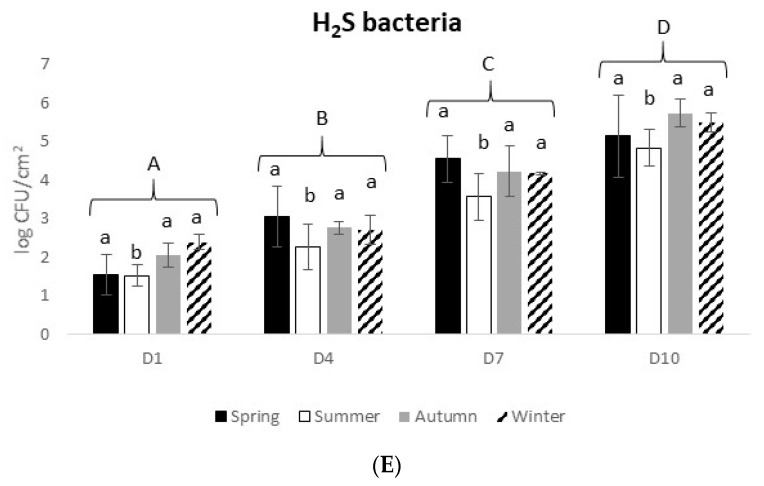
Seasonal Atlantic horse mackerel freshness profile over 10 days on ice, assessed by different methods: (**A**) Quality Index Method, (**B**) EC scheme, (**C**) Torrymeter (TRM), (**D**) microbiology (TVC), and (**E**) microbiology (H_2_S-producing bacteria). Values represent mean ± SD (*n* = 6). In Figure 3D,E, different uppercase letters indicate significant differences among days, while different lowercase letters indicate significant differences among seasons. In the other figures, different letters indicate significant differences among treatments.

For the wild species *T. trachurus*, the parameters measured by both sensory methods (QIM and EC scheme) presented a similar seasonal pattern as for the farmed species, also showing a decline in the freshness status with time in ice (Figure 3A,B). At D10, QIM ranged from 11 to 14.3 and the EC scheme ranged from 1.1 to 1.5. A significant interaction between time and seasons was observed for both methods (QIM, 2-way ANOVA on ranks, F_(9,80)_ = 4.61 and *p* < 0.0001; EC scheme, 2-way ANOVA on ranks, F_(9,80)_ = 8.99 and *p* < 0.0). The physical analysis also revealed a decline in the freshness profile of the fish with time, reaching values of 5–9.7 at D10, which were quite lower than those of the farmed species (Figure 3C). A significant interaction between factors was observed (2-way ANOVA on ranks, F_(9,80)_ = 2.20 and *p* < 0.03). Regarding the microbiological analysis, TVC were significant different between D1 and D10 (2-way ANOVA on ranks, F_(3,80)_ = 217.15 and *p* = 0.0), ranging from 10^5^ (D1) to 10^11^ cfu/cm^2^ (D10) (Figure 3D). For *T. trachurus*, the decline in TVC from the warmest seasons to the coldest ones was not evident, contrarily to the observedin farmed species. Only in summer was the number of colonies significantly lower than in the other seasons (2-way ANOVA on ranks, F_(3,80)_ = 4.39 and *p* = 0.006). Regarding the degradative bacteria, a significant increase in their number was observed with time (2-way ANOVA on ranks, F_(3,80)_ = 231.98 and *p* = 0.0), and significant differences among seasons were also observed (2-way ANOVA on ranks, F_(3,80)_ = 9.66 and *p* < 0.0001) (Figure 3E). In the winter, the number of degradative bacteria was significantly higher in *T. trachurus* than in the other farmed species.

#### 3.2.2. Atlantic Chub Mackerel

**Figure 4:**
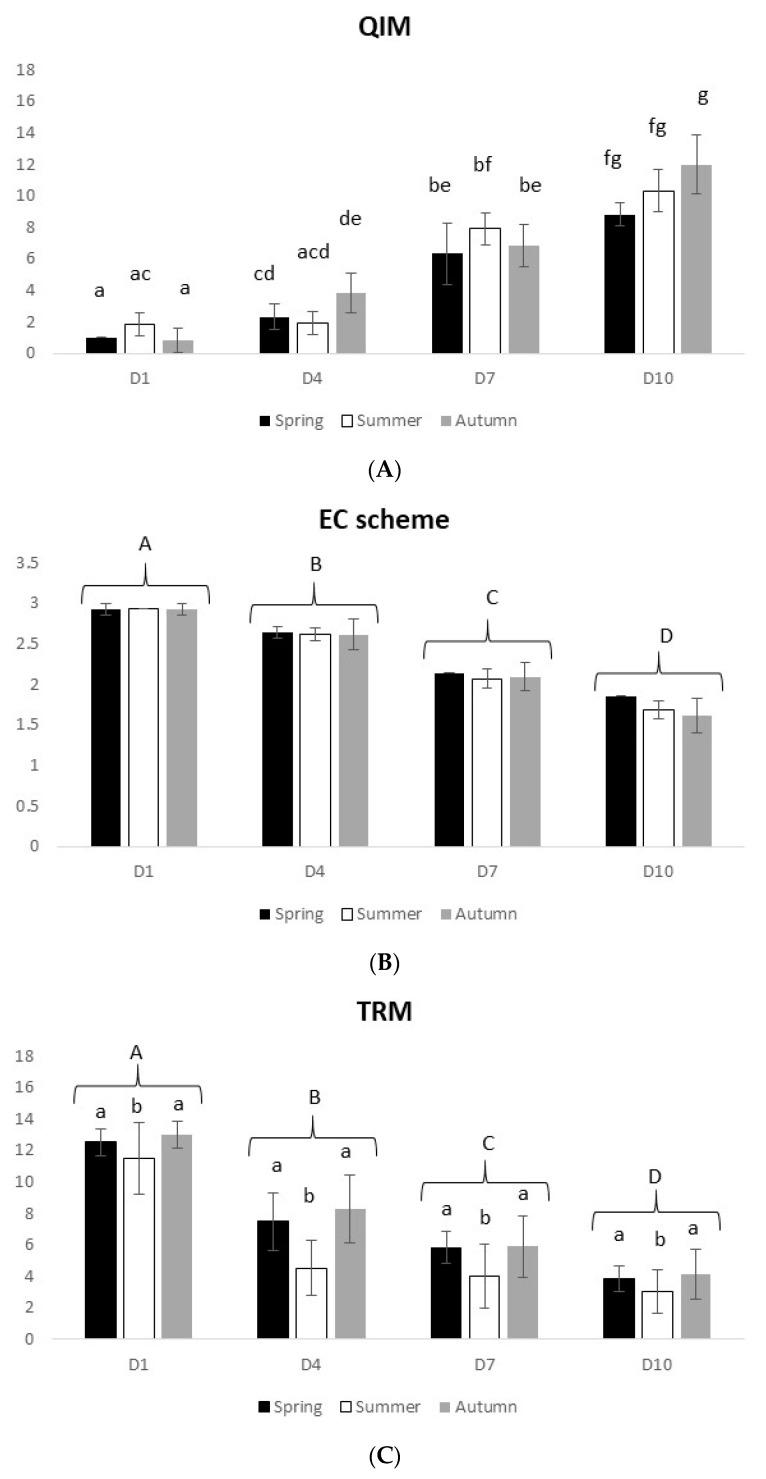

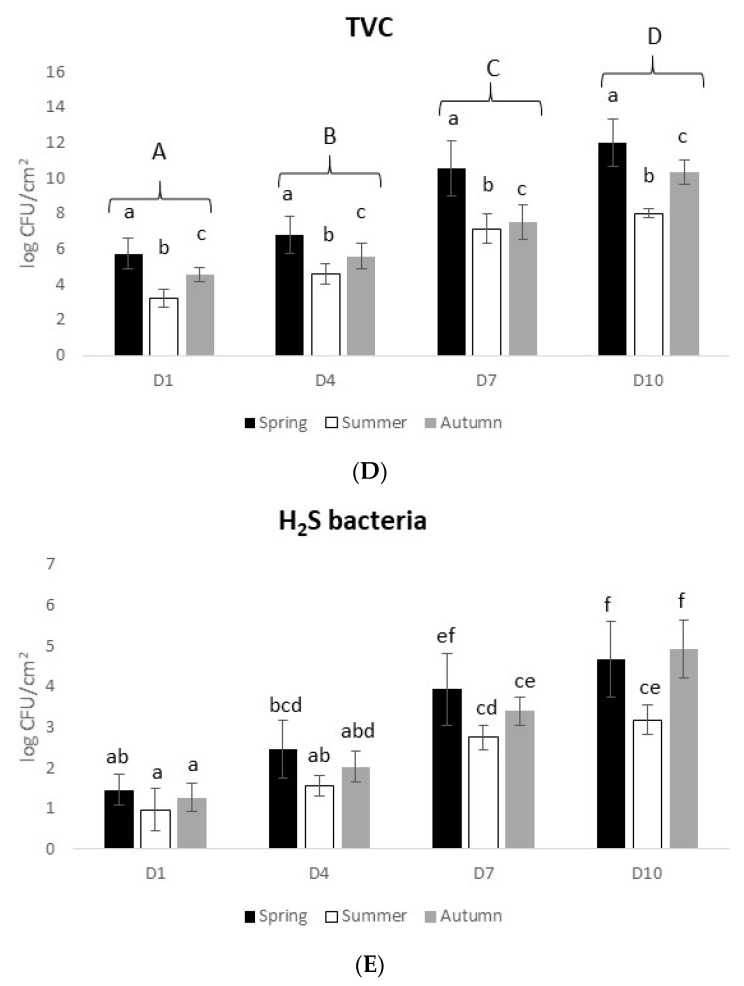
Seasonal Atlantic chub mackerel freshness profile over 10 days on ice, assessed by different methods: (**A**) Quality Index Method, (**B**) EC scheme, (**C**) Torrymeter (TRM), (**D**) microbiology (TVC), and (**E**) microbiology (H_2_S-producing bacteria). Values represent mean ± SD (*n* = 6). In Figure 4B–D, different uppercase letters indicate significant differences among days, while different lowercase letters indicate significant differences among seasons. In the other figures, different letters indicate significant differences among treatments.

The sensory analysis of *S. colias* revealed a clear decline of the freshness profile with time that was significant for the EC scheme method (2-way ANOVA on ranks, F_(3,60)_ = 366.8 and *p* = 0.0) (Figure 4B). For the QIM, a significant interaction between time and seasons was observed (2-way ANOVA on ranks, F_(6,60)_ = 3.81 and *p* = 0.003). At D10, QIM reached values of 8–12 and the EC scheme ranged from 1.6 to 1.8.

Regarding the physical analysis, there was a significant decline of electric impulses with time (2-way ANOVA on ranks, F_(9,80)_ = 4.61 and *p* < 0.0001) corresponding to a decline of fish freshness (Figure 4C). This species, in particular, at D10 reached the lowest TRM value (approximately 4) of the four studied species, indicating that it was close or even below the limit acceptable for consumption. Additionally, significant seasonal differences were observed, with spring and autumn presenting higher TRM values (higher freshness) (2-way ANOVA, F_(2,60)_ = 10.80 and *p* < 0.0001).

Concerning the microbiological analysis, the TVC followed the same pattern as for *T. trachurus*, showing an initial value of 10^5^ and significantly increasing until 10^12^ cfu/cm^2^ (D10) (2-way ANOVA on ranks, F_(3,60)_ = 134.74 and *p* = 0) (Figure 4D). Additionally, significant seasonal differences were observed (2-way ANOVA on ranks, F_(2,60)_ = 23.56 and *p* = 0). Concerning the degradative bacteria, a significant interaction between time and seasons was observed (2-way ANOVA on ranks, F_(6,60)_ = 2.31 and *p* < 0.045), with a tendency for an increase in the number of degradative bacteria with time being detected (Figure 4E).

#### 3.2.3. Sardine

The sardine species *S. pilchardus* was only available in two seasons, summer and autumn. As for the other species, a decline of freshness with the storage time was observed. These results were corroborated by all the used methods (see in detail Figure S1 of Supplementary Materials). For the sensory analysis, both QIM and EC scheme presented a significant interaction between factors (QIM, F_(3,16)_ = 11.29 and *p* < 0.05; EC scheme, F_(3,16)_ = 4.7 and *p* < 0.05). According to the physical and microbiological analyses, significant differences among sampling times (TRM, F_(3,16)_ = 102.8 and *p* < 0.05; TVC, F_(3,16)_ = 76.03 and *p* < 0.05; H_2_S bacteria, F_(3,16)_ = 73.95 and *p* < 0.05) and seasons (TRM, F_(1,16)_ = 6.67 and *p* < 0.05; TVC, F_(13,16)_ = 5.19 and *p* < 0.05; H_2_S bacteria, F_(1,16)_ = 27.61 and *p* < 0.05) were observed.

#### 3.2.4. PCO Ordinations

**Figure 5:**
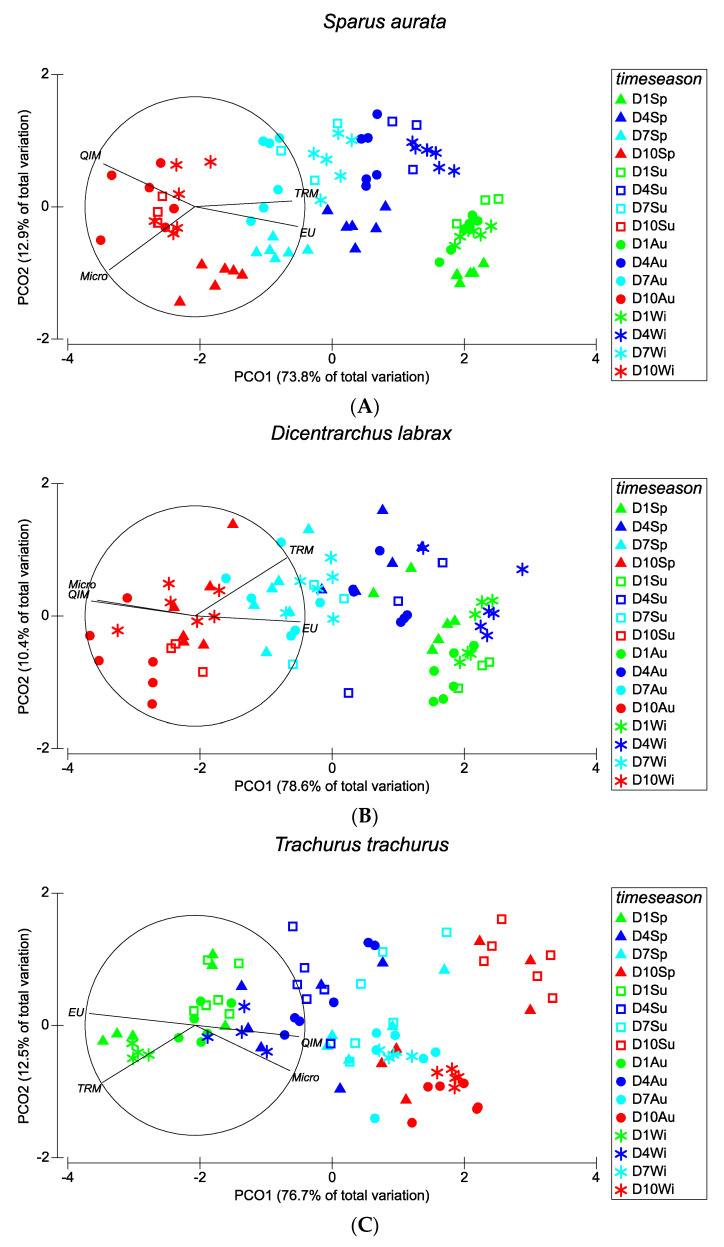

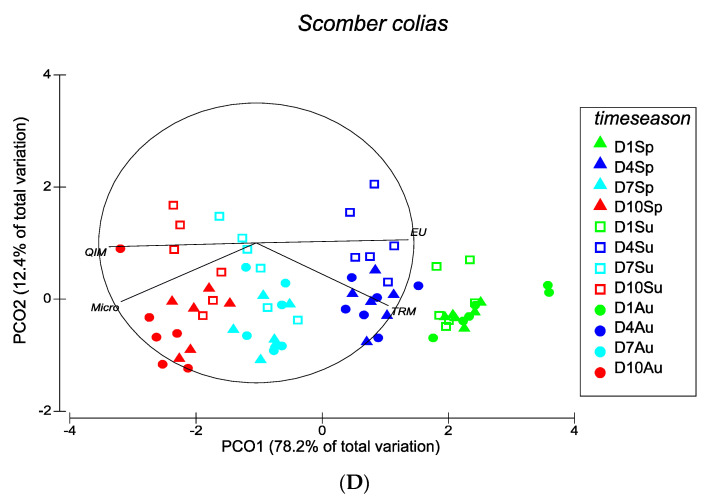
PCO ordinations for (**A**) *Sparus aurata*, (**B**) *Dicentrarchus labrax*, (**C**) *Trachurus trachurus*, and (**D**) *Scomber colias* assessed through four methods (QIM, EC scheme, TRM, and microbiology) for the different seasons over the 10 days of ice storage based on Spearman correlations. The length and direction of each vector indicate the strength and sign, respectively, of the relationship between the used methods and PCO axes. Sp—spring; Su—summer; Au—autumn; Wi—winter.

Multivariate analysis revealed that *S. aurata*, *D. labrax*, *T. trachurus*, and *S. colias* presented similar ordination patterns (Figure 5A–D), with a clear temporal separation of the freshness parameters from D1 to D10. Both TRM and the EC scheme were the parameters most responsible for the D1–D7 results, while QIM and microbiology were responsible for the lower freshness values observed at D10. For all the species, significant interactions between time and season were found (*S. aurata*, *D. labrax*, and *T. trachurus*, PERMANOVA, *p* (perm) = 0.001; *S. colias*, *p* (perm) = 0.002).

**Figure 6:**
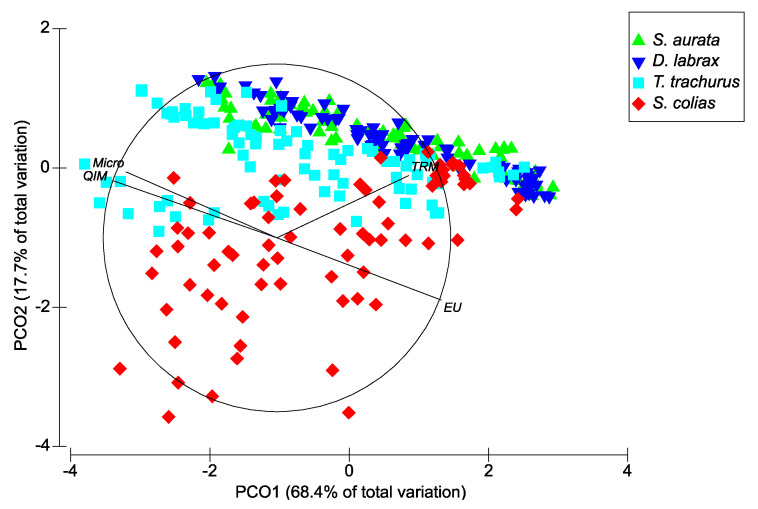
PCO ordination for the four studied fish species assessed with 4 methods (QIM, EU scheme, TRM, and microbiology) for the different seasons over the 10 days of ice storage based on Spearman correlations. The length and direction of each vector indicate the strength and sign, respectively, of the relationship between the used methods and the PCO axes.

When analyzing all the species together, it is clear that *S. aurata* and *D. labrax* were more similar between them and closer to *T. trachurus* than to *S. colias*. The latter species was the one that presented the greatest variability (Figure 6). Additionally, in this case, there was a significant interaction among the three factors (i.e., time, seasons, and species) (PERMANOVA, *p* (perm) = 0.001).

## 4. Discussion

Freshness is a parameter that is difficult to define despite being very common among consumers and all fish production chains [20]. The present data have revealed a clear temporal degradation of the quality of fish over storage time that was corroborated by all the used methods (QIM, EC scheme, TRM, and microbiology) for all the studied species. The findings obtained in the current study were in agreement with previous studies [11,13,21] for different fish species. However, it was not possible to make clear conclusions about seasonal differences in the freshness profile of most of the species, since significant interactions between time and seasons were found. Only for the seabass does it seem that higher freshness could be seen during winter, according to the physical and microbiological analyses. For the Atlantic chub mackerel, higher TRM values, and thus higher freshness, were recorded in the spring and autumn, whereas summer was a better month in terms of freshness according to the microbiological analysis. Thus, based on these results, it can be suggested that the best season to capture and commercialize seabass is the winter, while the best season for the Atlantic chub mackerel should be between spring and autumn. This is a pioneer study in terms of the seasonal evaluation of fish freshness, since other similar studies are almost nonexistent, which makes the comparison of these and other results quite difficult. In a recent study, Silva et al. [22] performed a seasonal sensory evaluation of five low commercial value or unexploited fish species. The results are difficult to compare with the current study. Nevertheless, Silva et al. [22] obtained significant seasonal differences in the sensory evaluation attributes. In addition, Durmus et al. [4] evaluated the effects of seasonal dynamics on sensory, chemical, and microbiological parameters of sardines and concluded that samples from winter deteriorated more rapidly than those from the spring season. Another work by Tzikas et al. [23] demonstrated the importance of seasonal variation in the chemical composition and microbiological condition of Mediterranean horse mackerel. According to this work, some seasonal variations on TVC and *Pseudomonas* spp. counts were observed, but no significant differences were observed for *S. putrefaciens*. These results were different from ours because no seasonal differences were detected for the Atlantic horse mackerel in the present study.

In addition, in the present work, *S. colias* revealed much lower TRM values than the other species, indicating a higher fragility of this species, which could be related to the fact that it is a fatty fish [24]. Species with a high lipid deposition are significantly more prone to autoxidation and rapid deterioration than lean fish, resulting in unstable food products [25]. The current study was in agreement with previous ones that demonstrated greater variation in TRM and a faster decline of slope in fatty fish, indicating that the properties being assessed were changing more rapidly in species with high lipid contents [26].

The freshness data obtained in this study were also supported by microbiological studies. In live fish, microorganisms are mainly associated with their outer or external surface (with the exception of the gastrointestinal tract), such as the skin, mucus, and gills [27]. As such, the microbial load of outer surface fish tissues could be valid information related to fish freshness, as microbial growth is intensified sometime after fish death. Within the degradative flora of fish, sulfide (H_2_S)-producing bacteria are considered an adequate indicator of fish spoilage [28]. In the present study, at sampling day 1 (D1), all analyzed fish species presented H_2_S-producing bacteria in their skin, with numbers ranging between 10^1^ and 10^2^ cfu/cm^2^—three times lower than the TVC. After 10 days of ice storage, the numbers of H_2_S-producing bacteria increased by three-fold. This was an expected result since these microorganisms are usually prevalent in the microbial flora responsible for fish spoilage, with *Shewanella putrefaciens* being the most common food spoiler bacteria of this group [29]. In general, the percentage of degradative bacteria corresponded to 30–40% of total viable counts, which means that the majority of bacteria corresponded to species other than H_2_S-producing bacteria.

Additionally, data obtained in the current study showed that farmed species that reach day 10 of storage time presented lower values of QIM and microbial counts (cfu) and higher values of TRM compared with the wild species. This makes sense since farmed fish can frequently reach retailers and consumers in a shorter time than wild ones. Additionally, the fish-catching process and handling practices associated with wild fisheries are factors that can compromise the quality of fish.

On the other hand, TVC were lower in farmed species than in wild fish; in the former, a decline in numbers was detected from spring/summer to autumn/winter, while in the latter, this was not clear. This could be related to the fact that in aquaculture systems, the fluctuation of temperatures in the tanks between the warmest and coldest seasons can be higher than in the open sea. These environmental conditions are known to influence the microbial community of fish.

These results reinforced the idea that farmed fish can have high quality/freshness, despite there are some consumers that prefer wild fish over farmed options because they assume that the latter has a lower quality [30]. A previous study from Engle et al. [31] found that farmed fish are usually fresher at purchase, possibly because of the greater control of their production and distribution. Thus, these results can also contribute to improving the image of farmed fish.

## 5. Conclusions

The methods used in this work showed an unclear relationship between the freshness status of the fish and the season. However, when taken all together, they indicate a loss of freshness that gradually occurs over time. Though one could expect differences in freshness induced by season, as different seasons imply differences in many parameters (e.g., oxygen, pH, and temperature—the last being the most important one), it seems that the effect of seasonality was not as assertive as expected, at least for the majority of the studied species. However, for seabass and Atlantic chub mackerel, some indications for the acquisition of fresher individuals in winter and spring–autumn, respectively, can be given. The handling and preservation methods have been shown to be effective in slowing down freshness loss, resulting in less detectable differences in final product quality. It can also be admitted that handling and preservation methods in industry, and even in laboratories, are not ideal or perfect, so freshness could be even more preserved if improvements (in temperature and hygiene, for example) are introduced to distribution chains. Nonetheless, in any case, it seems that the time of the year (season) does not clearly influence the freshness status of fresh fish distribution for both the wild and farmed species tested in this study. Interestingly, farmed species have shown signs of high freshness, which is favorable to the consumption of cultivated fish.

## Supplementary Materials

The following are available online at https://www.mdpi.com/article/10.3390/foods10071567/s1, Figure S1: Seasonal sardine freshness profile during 10 days on ice, assessed by different methods: (A) Quality Index Method; (B) EC scheme; (C) Torrymeter (TRM); (D) Microbiology (TVC); (E) Microbiology (H2S bacteria). Values represent mean ± SD (summer: *n* = 6; autumn: *n* = 3). In Figure S1C–E, uppercase letters indicate significant differences among days, while lowercase letters indicate significant differences among seasons. In the other figures different lowercase letters indicate significant differences among treatments.
