# Supplementary material for: Seasonal Evaluation of Freshness Profile of Commercially Important Fish Species
Source: Foods. 2021 Jul 6;10(7):1567. doi: 10.3390/foods10071567 (PMC8307230; doi:10.3390/foods10071567)
Supplement: Supplementary file 1 [file foods-10-01567-s001.zip › foods-1239998-supplementary.pdf]

Sardine

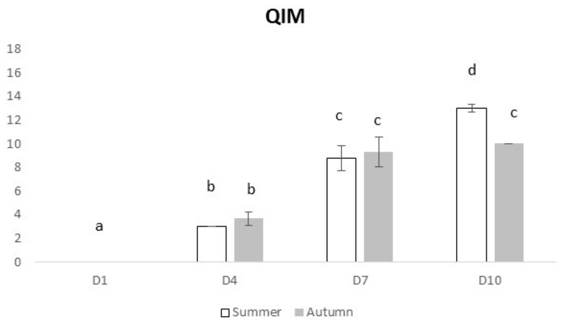
QIM
18
16
14
12
10
8
6
4
2
0
a
b
b
c
c
d
c
D1
D4
D7
D10
Summer
Autumn

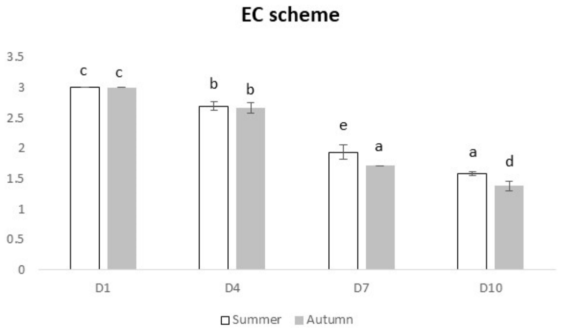
EC scheme
3.5
3
2.5
2
1.5
1
0.5
0
c
c
b
b
e
a
a
d
D1
D4
D7
D10
Summer
Autumn

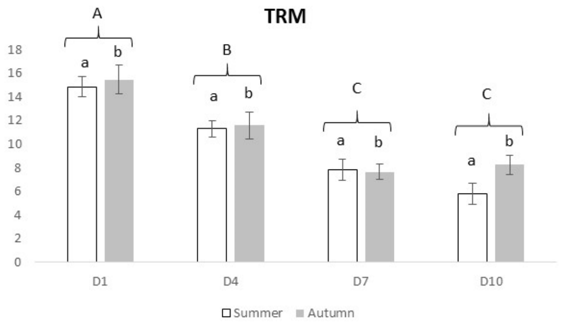
TRM
A
B
C
C
18
16
14
12
10
8
6
4
2
0
a
b
a
b
a
b
a
b
D1
D4
D7
D10
Summer
Autumn

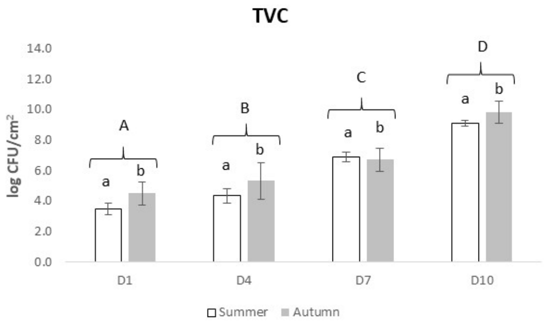
TVC
log CFU/cm2
14.0
12.0
10.0
8.0
6.0
4.0
2.0
0.0
A
B
C
D
a
b
a
b
a
b
a
b
D1
D4
D7
D10
Summer
Autumn

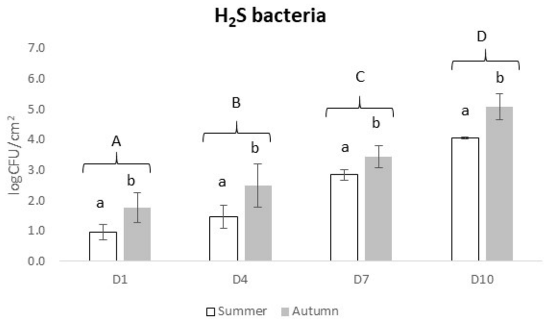

Figure S1 - Seasonal sardine freshness profile during 10 days on ice, assessed by different methods: A) Quality Index Method; B) EC scheme; C) Torrymeter (TRM); D) Microbiology (TVC); E) Microbiology (H_2S bacteria). Values represent mean ± SD (summer: n = 6; autumn: n = 3). In figs S1 C, D and E, uppercase letters indicate significant differences among days, while lowercase letters indicate significant differences among seasons. In the other figures different lowercase letters indicate significant differences among treatments.
